# Uretero‐arterial fistula treated with endovascular stent graft following multiple interventions

**DOI:** 10.1002/iju5.12216

**Published:** 2020-08-30

**Authors:** Tomoyuki Kaneko, Akihiko Sakamoto, Yukio Yamada, Masayoshi Yamamoto, Hiroshi Kondo, Tohru Nakagawa

**Affiliations:** ^1^ Department of Urology Teikyo University School of Medicine Tokyo Japan; ^2^ Department of Radiology Teikyo University School of Medicine Tokyo Japan

**Keywords:** angiography, nephrectomy, stents, ureter, vascular fistula

## Abstract

**Introduction:**

Uretero‐arterial fistula is a rare life‐threatening condition. Its diagnosis and management remains a challenge for urologists.

**Case presentation:**

A 64‐year‐old man presented to our hospital with gross hematuria. He had history of rectal cancer treated with neoadjuvant chemoradiotherapy followed by low anterior resection and chronic ureteral stenting for bilateral ureteral strictures. He developed recurrent hemorrhagic shocks due to sudden massive gross hematuria. Repeated computed tomography and angiography could not identify the source of bleeding. After prophylactic embolization of the right renal artery and right nephrectomy, angiography finally revealed a uretero‐arterial fistula from the right external iliac artery. Percutaneous balloon‐expandable covered stent graft was used to successfully treat the fistula.

**Conclusions:**

Urologists should consider possible uretero‐arterial fistula in patients with recurrent hematuria along with several risk factors and convincing medical history.

**Abbreviations & Acronyms**
CT: computed tomography
UAF: uretero‐arterial fistula

**Keynote message**

UAF is a rare yet life‐threatening condition. Its diagnosis and management remains a challenge for urologists. Urologists should take into account possible UAF in patients with recurrent hematuria along with several risk factors and convincing medical history.

## Introduction

UAF is a rare condition which was first reported in 1908 by Moschcowitz.^1^ It can be a life‐threatening condition, owing to potential massive blood loss. Due to its rarity, the diagnosis and management of UAF remains a challenge for urologists. Some studies suggested that the incidence of UAF increased in recent years, presumably due to the improvement in the prognosis of patients with chronic ureteral stents.^2^ Nonetheless, UAF would be a rare condition that many urologists encountered once or never in their career. We present a case of UAF treated with endovascular intervention following multiple angiographic procedures and nephrectomy.

## Case presentation

**Fig. 1:**
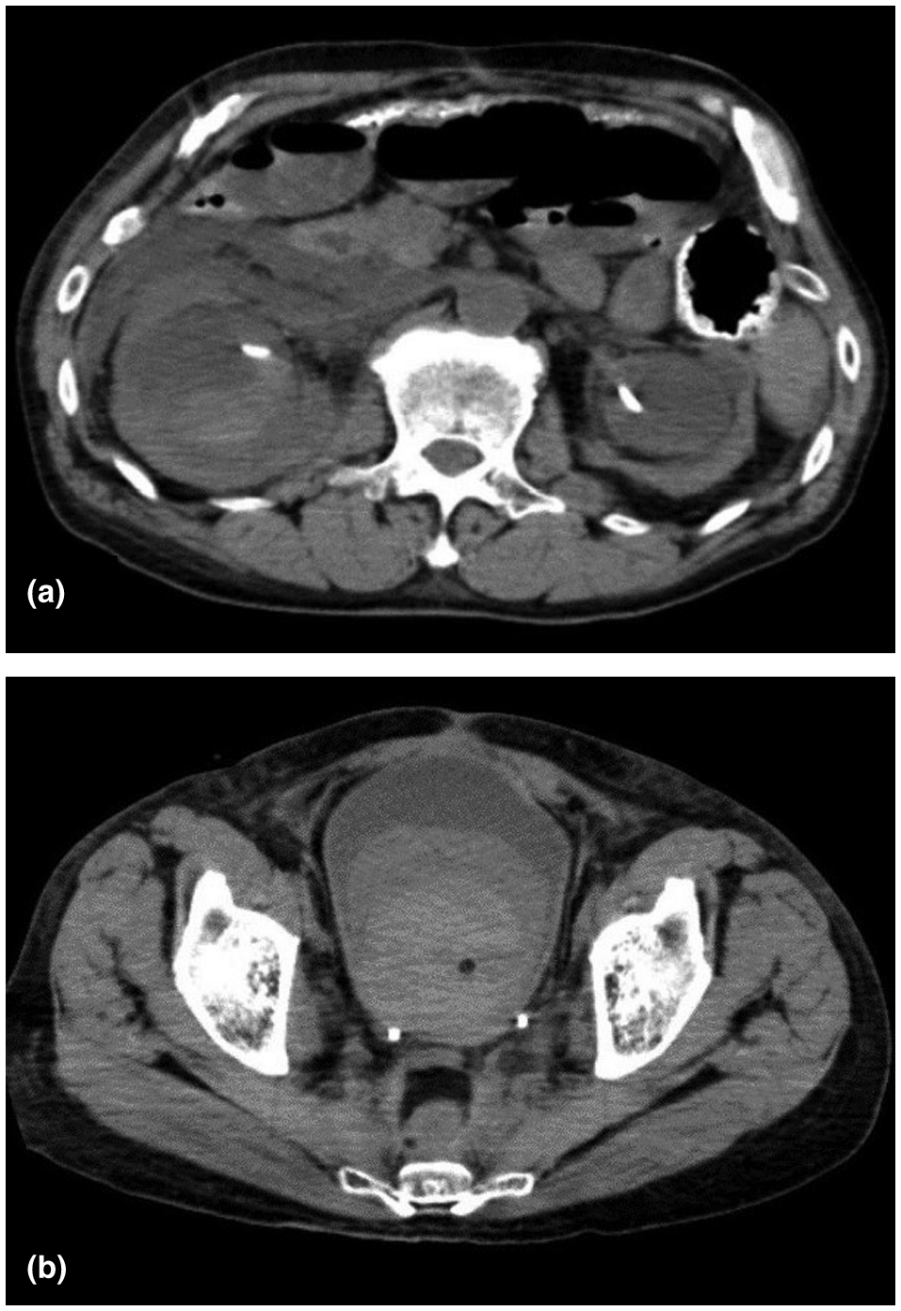
Non‐contrast‐enhanced CT shows hematoma in the right renal pelvis (a) and bladder (b).

**Fig. 2:**
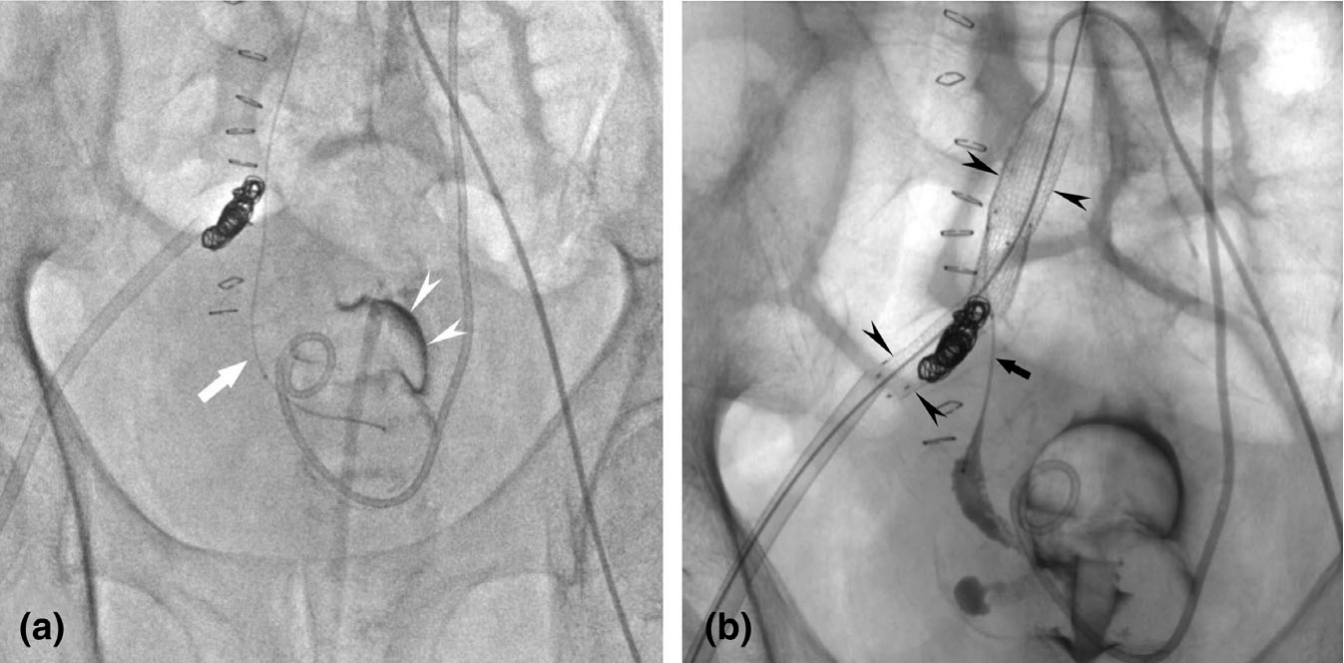
(a) Digital subtraction angiography demonstrated a contrast material (arrowheads) flowing into the urinary bladder after injection through a microcatheter (arrow) within the right ureter and urinary bladder. (b) Fluoroscopic image shows two stent grafts (arrowheads) in the right common iliac artery and selective cannulation of the right ureter (arrow) from UAF.

A 64‐year‐old man was brought to the emergency department of our hospital due to gross hematuria and high fever. He was diagnosed with septic shock and, thereafter, admitted to the intensive care unit and intubated. Non‐contrast‐enhanced CT of the abdomen revealed hematoma in the right renal pelvis and bladder (Fig. 1a,b). In parallel with intensive care, bladder hematoma was cleared, and continuous bladder irrigation commenced. He had a history of rectal cancer and received neoadjuvant chemoradiotherapy followed by low anterior resection 12 years before the presentation. He developed acute renal failure due to bilateral ureteral strictures presumably caused by radiation 8 years before the presentation. Subsequently, he underwent bilateral ureteral stenting and regularly visited our hospital for stent exchange every 3 months. Ten days after the admission, he developed hemorrhagic shock due to a sudden massive gross hematuria. On suspicion of a right UAF, emergency angiography was performed; however, it was not able to identify the source of bleeding. Prophylactic embolization of the right renal artery was executed on an equivocal suspicion of renal hemorrhage. Eighteen days after the admission, he again developed hemorrhagic shock due to a sudden massive gross hematuria for the second time. However, contrast‐enhanced CT and angiography could not identify the source of bleeding either. He underwent prophylactic right nephrectomy. Severe adhesion due to prior abdominal surgery, inflammation, and radiotherapy resulted in major bleeding and precluded dissection of the right distal ureter. Twenty‐eight days after the admission, he developed hemorrhagic shock for the same massive gross hematuria again for the third time. Contrast‐enhanced CT suggested a small pseudoaneurysm in the right external iliac artery at the iliac‐ureteral crossover. Based on UAF diagnosis, he underwent emergency angiography and endovascular treatment. Under local anesthesia, the 4‐Fr angiographic sheath was inserted via the left femoral artery. An angled guidewire and angiographic catheter were advanced across the aortic bifurcation into the right common iliac artery. UAF was confirmed by angiograms. Coil embolization of the right proximal internal iliac artery was performed to secure an adequate distal landing zone for stent graft placement. Direct probing of the ureter was performed with a hydrophilic guidewire and microcatheter under fluoroscopic guidance (Fig. 2a). Percutaneous balloon‐expandable covered stent graft (Viabahn^®^; Gore, Flagstaff, AZ, USA) was used to treat the fistula. The 12‐Fr angiographic sheath was placed through the right femoral artery. After systemic heparinization with a bolus dose of 3000 U, two stent grafts (distal stent, 8 × 50 mm; proximal stent, 10 × 50 mm) were deployed to cover the entire lesion (Fig. 2b). Occlusion of the ureteral segment using n‐butyl cyanoacrylate was performed subsequently. The final angiogram confirmed the appropriate placement of stent grafts at the target lesion. The patient had no further episodes of gross hematuria after the endovascular treatment. However, he went on chronic hemodialysis due to an exacerbation of chronic kidney disease. He also suffered from disuse syndrome resulting from multiple intensive therapy. Furthermore, he developed an adjustment disorder and anorexia. Seven months after the endovascular treatment, he died of acute respiratory failure due to massive transudative pleural effusion caused by malnutrition.

## Discussion

UAF is a rare condition with only approximately 150 cases reported in literature.^2^ Due to its scarcity, the diagnosis of UAF can often be delayed or missed. Massive blood loss owing to UAF is sometimes life‐threatening. Several studies have reported a mortality rate of 7–23% and was strongly related to diagnostic delay.^3^, ^4^ The main presenting symptom of UAF is gross hematuria, which is sometimes the only symptom as well. The risk factors of UAF are referred to as a clinical triad: chronic ureteral stenting, abdominal or pelvic surgery, and pelvic radiotherapy.^2^ Vascular diseases, such as iliac artery aneurysms, are some of the other risk factors.^2^, ^5^ The diagnostic procedures of UAF is challenging. Although contrast‐enhanced CT is a noninvasive and useful diagnostic device, its sensitivity is low.^2^, ^5^ Digital subtraction angiography is the best modality for UAF diagnosis. However, angiography may not show abnormal findings in approximately one‐third of patients with UAF.^2^ A systematic review of 139 cases demonstrated that 2.4 diagnostic measures per patient were performed before a definitive diagnosis of UAF on average.^5^ CT and angiography were helpful in only 42% and 69% of cases respectively.^5^ Of all 246 treatments described, 19 unsuccessful treatments were carried out based on incorrect diagnosis, including nephrectomy in 11 and renal arterial embolization in four patients.^5^ Several case series reported the efficacy of provocative angiography, which involves simultaneous angiography and transurethral intervention of rubbing ureteral lumen by balloon catheter in the ureter.^6^ Such maneuvers may induce significant hemorrhage. Therefore, it should only be done by a well‐trained multidisciplinary medical team.^7^ Historically, the treatment of UAF was open surgical repair. However, surgical intervention in patients with UAF is often difficult because of hemodynamic instability resulting from massive hemorrhage and hostile abdomen from prior surgery and radiotherapy. Therefore, endovascular treatment with stent graft has become the mainstay in the management of UAF in recent years.^5^, ^8^ UAF should be included in the differential diagnosis of patients with unexplained hematuria who have a history of chronic ureteral stenting, pelvic surgery, and radiotherapy. Furthermore, it would be reasonable to intervene without definitive imaging evidence in patients possessing several risk factors and convincing medical history.

## Conflict of interest

The authors declare no conflict of interest.

## References

[1] Moschcowitz AVIX . Simultaneous ligation of both external iliac arteries for secondary hemorrhage. Ann. Surg. 1908; 48: 872–5. 17862274 10.1097/00000658-190812000-00009 PMC1407034

[2] Pillai AK , Anderson ME , Reddick MA , Sutphin PD , Kalva SP . Ureteroarterial fistula: diagnosis and management. AJR Am. J. Roentgenol. 2015; 204: W592–8. 25905967 10.2214/AJR.14.13405

[3] Bergqvist D , Pärsson H , Sherif A . Arterio‐ureteral fistula–a systematic review. Eur. J. Vasc. Endovasc. Surg. 2001; 22: 191–6. 11506509 10.1053/ejvs.2001.1432

[4] van den Bergh RC , Moll FL , de Vries JP , Yeung KK , Lock TM . Arterio‐ureteral fistula: 11 new cases of a wolf in sheep's clothing. J. Urol. 2008; 179: 578–81. 18078959 10.1016/j.juro.2007.09.087

[5] van den Bergh RC , Moll FL , de Vries JP , Lock TM . Arterioureteral fistulas: unusual suspects‐systematic review of 139 cases. Urology 2009; 74: 251–5. 19362353 10.1016/j.urology.2008.12.011

[6] Vandersteen DR , Saxon RR , Fuchs E , Keller FS , Taylor LM Jr , Barry JM . Diagnosis and management of ureteroiliac artery fistula: value of provocative arteriography followed by common iliac artery embolization and extraanatomic arterial bypass grafting. J. Urol. 1997; 158: 754–8. 9258074

[7] Hirsch LM , Amirian MJ , Hubosky SG , Das AK , Abai B , Lallas CD . Urologic and endovascular repair of a uretero‐iliac artery fistula. Can. J. Urol. 2015; 22: 7661–5. 25694016

[8] Guntau M , Hegele A , Rheinheimer S , Hofmann R , Mahnken AH . Balloon‐expandable stent graft for treating uretero‐iliac artery fistula. Cardiovasc. Intervent. Radiol. 2017; 40: 831–5. 28150018 10.1007/s00270-017-1586-4

